# A meta-synthesis of the transitioning experiences and career progression of migrant African nurses

**DOI:** 10.1186/s12912-023-01273-1

**Published:** 2023-04-06

**Authors:** Jonathan Bayuo, Mary Abboah-Offei, Precious Adade Duodu, Yakubu Salifu

**Affiliations:** 1 Department of Nursing and Midwifery, Presbyterian University, Kwahu East, Ghana; 2 School of Health and Social Care, Edinburgh Napier University, Edinburgh, UK; 3 Department of Nursing and Midwifery, School of Human and Health Sciences, University of Huddersfield, Queensgate, Huddersfield, England, UK; 4 Division of Health Research, Faculty of Health and Medicine, Lancaster University, Lancaster, UK

**Keywords:** Migrant African nurses, Nursing shortage, Global migration, Transition experiences, Career progression, Meta-synthesis

## Abstract

**Introduction:**

With the rise in global migration, hospitals and health systems in developed countries are looking to supplement their workforces with migrant nurses who have been reported to feel devalued, underutilized with experience of deskilling and unmet expectations as they transitioned. Despite the plethora of literature reporting on the experiences of internationally trained nurses, only limited work has been done regarding understanding the experiences of Migrant African nurses. Thus, this study sought to synthesize existing qualitative studies to develop in-depth understanding of the transitioning experiences of migrant African nurses, their career progression and to highlight existing gaps to guide future studies as well as inform policies.

**Method:**

A meta-synthesis was performed and reported according to the Preferred Reporting Items for Systematic Reviews and Meta-Analyses and the Enhancing transparency in reporting the synthesis of qualitative research statement. A pre-planned search strategy was developed guided by the SPIDER tool for qualitative synthesis searching EMBASE via OVID, CINAHL via EBSCO, PubMed, Web of Science, and PsychINFO databases. We included published studies that 1) focused on migrant African nurses, 2) employed a qualitative design and 3) reported in English.

**Results:**

The search yielded 139 studies of which nine studies met the inclusion criteria and included in final synthesis. Three themes with corresponding subthemes emerged from data synthesis: 1) Navigating reality shock (a. Navigating a new culture, b. Survival strategies and support amidst the shock); 2) Discrimination and limited opportunities for promotion (a. Prejudices and preference for White over Black, b. Lack of recognition and limited opportunities for a workplace promotion); and 3) Finding one’s feet (a. Standing up for oneself and looking beyond discrimination, b. Experiencing growth).

**Conclusion:**

Transitioning to a new setting can be a challenging experience for migrant African nurses warranting the availability of a tailor-made adaptation or orientation programme. Though African nurses may experience discrimination and prejudices as part of their transition, they consider their situation to be better off compared to back home. Therefore, clear transitioning policies which focus on career pathways are required by hiring institutions, and migrant nurses should be proactive in taking active roles in pushing their career ahead, instead of maintaining a culture of silence.

## Background

Changing demographics, an increasingly ageing population, and increased demands for healthcare have contributed to an ongoing global nursing shortage [1]. According to the International Centre on Nurse Migration (ICNM) approximately 10.6 million new nurses will be needed to address the existing nurse shortage over the next decade and to replace the 4.7 million nurses who are expected to retire [2].

Amidst the nursing shortage is an ongoing nurse migration with most nurses moving from developing to developed or industrialized settings [3]. The migration of nurses across the globe is not a new phenomenon [1, 3, 4]. In fact, several countries now depend on internationally trained nurses to meet domestic shortages [5]. This pattern has however been exacerbated during the corona virus disease (COVID-19) pandemic and is projected to escalate further in the post-pandemic era [6].

In sub-Sahara Africa, nurses make up a critical component of the health workforce in terms of patient care [7]. This may be related to the shortage of physicians which creates a gap for nurses to support patient care delivery at all levels [7, 8]. In fact, nurses keep the health system running [7], and as such their migration from the continent may represent a significant loss of human resources to the respective countries [9–12]. A recent cross-sectional study across 47 African countries reported that the total stock of health workers was approximately 3.6 million which was inadequate representing a shortage across these countries [13].

Although there is currently limited primary research regarding migration motivation among migrant African nurses (herein referred to as nurses migrating from any country in the African continent) [14, 15], findings from existing reports highlight several potential factors that may contribute to the phenomenon including low salaries, poor working conditions, lack of continuing professional development opportunities, heavy workload, low job satisfaction, and lack of resources [12, 14–17]. An old, extant study has highlighted that whereas African nurses are motivated by economic factors and career progression to migrate, nurses from developed settings such as Australia, Canada, and New Zealand are usually motivated by travel to migrate [18]. In another study, the authors observed that foreign-trained nurses are often motivated to migrate to the UK to experience another culture, become exposed to professional development and attain more financial rewards [19].

Irrespective of the migration motivation, internationally recruited nurses undergo a transitioning process to adapt to their new settings which is often a complex phenomenon [20–22]. migrant African nurses have been reported to perceive workplace discrimination, racism, and a general lack of support as they transitioned to their new settings and clinical roles [23–28]. A more recent study noted that migrant nurses transitioning to the Australian system also experienced loneliness, discrimination, and felt incomplete without their families [29]. Also, migrant nurses have been reported to feel devalued, underutilized, experience deskilling or credentialing issues, disappointment, and unmet expectations as they transitioned [20, 30, 31]. In addition to the often chaotic transitioning experiences, a recent systematic review reported that migrant nurses are at a high risk of work-related injuries and discrimination than native nurses [32]. Thus, migrant nurses may face challenging transitioning and integration processes which may impact their long-term stay in a setting [33]. Despite the potentially negative experiences, some earlier studies observed that some migrant nurses may have positive transitioning experiences, particularly if they underwent an adaptation programme [34, 35].

Beyond their transitioning experiences, the career progression of migrant nurses remains another critical issue worth mentioning. In 2009, this issue was raised among stakeholders in the Irish healthcare system as only a few migrant nurses had achieved managerial grades in Ireland (Clinical Nurse Manager 1[CNM1] or Clinical Nurse Manager 2 [CNM2]), noting that perhaps this stemmed from their reluctance to apply for senior post [36]. A study that included migrant nurses working in Asia and Middle East also highlighted their stalled career progression though they fulfilled all requirements [37]. Additionally, a recent systematic review and qualitative meta-synthesis that compared foreign-trained dentists, nurses, and doctors in the UK highlighted that nurses reported a wider knowledge and skills gap, more multi-level discrimination and less career progression compared to the doctors [38]. Foreign-trained nurses in another study described racism as a significant issue adversely impacting their career progression [30]. Notwithstanding these findings, migrant African nurses remain underrepresented in most of these studies and their transitioning experiences and career progression remain poorly articulated in existing literature despite the reality of ongoing nurse migration from Africa [14, 23, 28, 39].

The increasing number of nurses migrating from Africa to other countries warrants an understanding of how they transition to a new setting and their career progression pathways. Studies have called for more support to enhance the successful integration of international nurses into the healthcare systems of these countries and the wider society [24, 25, 28, 33]. This is particularly important as negative experiences can impact retention [5]. Determining the forms of support require an in-depth understanding of their experiences as they transition to their new settings and progress on their career pathways.

Consequently, the issues explored in this study represents an area requiring further research. It is evident that despite much attention to the workplace integration of internationally educated/trained nurses into to developed countries, major cultural adjustments exist for both employees and employers. It is also noted that nurses from African countries have historically experienced greater challenges. This research examined and shed light on key areas and potential for reform. The current study, therefore, sought to synthesize existing qualitative studies to develop in-depth understanding of the transitioning experiences of migrant African nurses as well as their career progression and highlight existing gaps to guide future studies as well as inform policies.

## Methods

### Study design

A qualitative meta-synthesis was undertaken [40]. Qualitative meta-synthesis is a method of interpreting and synthesising qualitative findings across individual studies to develop a deeper understanding of a phenomenon which makes it appropriate for the current study [40, 41]. Meta-synthesis entails the authors’ interpretations of the primary data by the original authors [40, 42], and therefore, presents a high level synthesized data. This qualitative meta-synthesis is reported according to the Preferred Reporting Items for Systematic Reviews and Meta-Analyses (PRISMA) [43] and the Enhancing transparency in reporting the synthesis of qualitative research (ENTREQ) statement [44]. A study protocol was developed to guide the conduct of this meta-synthesis but was not published.

### Search strategy

A pre-planned search strategy was developed with the assistance of a librarian. An initial limited search was undertaken in EMBASE and CINAHL following which a comprehensive search strategy was developed guided by the SPIDER tool for qualitative synthesis [45]. The search strategy that was formulated based on the SPIDER tool are as follows: Sample (migrant African nurses); Phenomenon of Interest (transitioning experiences); Design (interviews and focus group discussions); Evaluation (experiences); and Research type (qualitative studies, and multi-method or mixed method with qualitative data reported).

The full search sources were undertaken from the inception of the database to 31^st^ August 2022 in the following databases: EMBASE via OVID, CINAHL via EBSCO, PubMed, Web of Science, and PsychINFO. The reference sections of identified studies were also hand searched for potential studies. The search terms used were ‘nurses’ or ‘nursing staff’ or ‘nurse’ or ‘nursing’ AND ‘migrants’ or ‘immigrants’ AND ‘transition’ or ‘transitions’ or ‘transitioning’ AND ‘career progression’ or ‘promotion’ or ‘career advancement’ or ‘career development’. The World Health Organization’s list of African countries were used to guide the search for potential studies. Only peer-reviewed qualitative studies containing verbatim quotes relevant to the transitioning experiences and career progression of migrant African nurses were considered eligible for inclusion in this study. In this meta-synthesis, qualitative studies were defined as those using methodologies such as grounded theory, phenomenology (descriptive or hermeneutic), ethnography, interpretive description, and qualitative description.

### Study screening and selection

**Fig. 1:**
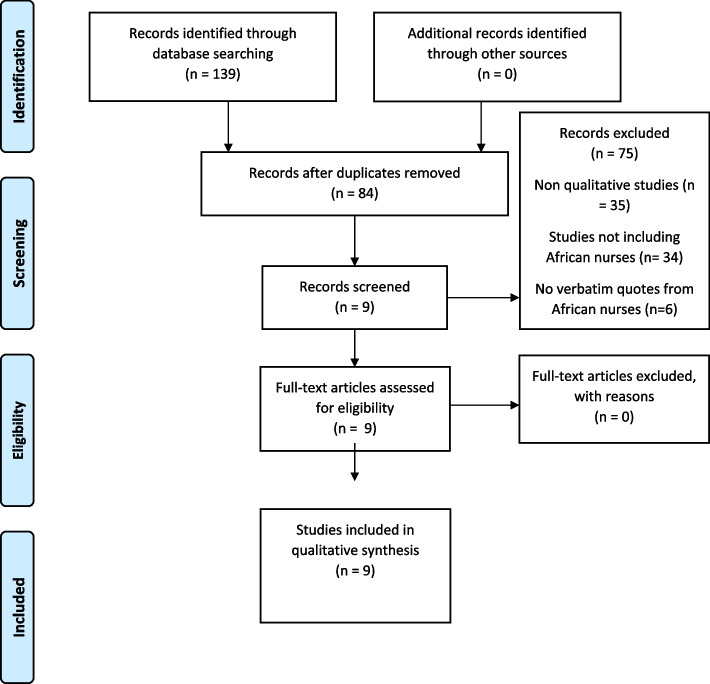
PRISMA Flowchart of Study Selection

References of all identified studies were exported into Endnote X9.2 following which de-duplication was undertaken by the authors. Title and abstract screenings were independently undertaken by the authors. The inclusion criteria were: 1) published studies that focused on migrant African nurses, 2) employed a qualitative design (as described above), and 3) reported in English. Qualitative studies that included other migrant nurses were considered for inclusion if they specified the verbatim quotes of the African participants. Considering the nature of the review, no publication year limits were applied. Mixed method studies were considered for inclusion if they provided qualitative data relating to transitioning experiences of migrant African nurses. Preprints, unpublished thesis, and grey literature were excluded. The results of the search are presented in the PRISMA flow diagram presented in Fig. 1.

### Appraisal of methodological quality

The Joanna Briggs Institute (JBI) 10-item standardized critical appraisal checklist for qualitative studies was used to critically appraise included studies (see Table 1 for appraisal results). As reported in a previous meta-synthesis, a minimum of ‘yes’ for six domains was required for inclusion [46].

**Table 1:** Critical appraisal

Authors	Is there congruity between the stated philosophical perspective and the research methodology?	Is there congruity between the research methodology and the research question or objectives?	Is there congruity between the research methodology and the methods used to collect data?	Is there congruity between the research methodology and the representation and analysis of data?	Is there congruity between the research methodology and the interpretation of results?	Is there a statement locating the researcher culturally or theoretically?	Is the influence of the researcher on the research, and vice- versa, addressed?	Are participants, and their voices, adequately represented?	Is the research ethical according to current criteria or, for recent studies, and is there evidence of ethical approval by an appropriate body?	Do the conclusions drawn in the research report flow from the analysis, or interpretation, of the data?	Overall Appraisal
Aboderin, 2007 [47]	Yes	Yes	Yes	Yes	Yes	Yes	Unclear	Yes	Yes	Yes	Include
Alexis et al., 2007 [48]	Yes	Yes	Ye	Yes	Yes	Yes	Unclear	Yes	Yes	Yes	Include
Henry, 2007 [49]	Yes	Yes	Yes	Yes	Yes	Yes	Unclear	Yes	Yes	Yes	Include
Iheduru-Anderson & Wahi, 2018 [50]	Yes	Yes	Yes	Yes	Yes	Yes	Unclear	Yes	Yes	Yes	Include
Jose, 2011 [51]	Yes	Yes	Yes	Yes	Yes	Yes	Unclear	Yes	Yes	Yes	Include
Larsen, 2007 [52]	Yes	Yes	Yes	Yes	Yes	Yes	Unclear	Yes	Yes	Yes	Include
Likupe, 2015 [26]	Yes	Yes	Yes	Yes	Yes	Yes	Unclear	Yes	Yes	Yes	include
Likupe & Archipong, 2013 [27]	Yes	Yes	Yes	Yes	Yes	Yes	Unclear	Yes	Yes	Yes	Include
Okougha & Tilki, 2010 [53]	Yes	Yes	Yes	Yes	Yes	Yes	Unclear	Yes	Yes	Yes	Include

### Data extraction and synthesis

Standard information including authors, settings, study aims, methods employed, and sample sizes were extracted from the included studies. An inductive analytical approach inspired by Ricoeur’s hermeneutics was employed to analyse and interpret the quotes [54]. The inductive analytical strategy proceeded through naive reading, thematic structural analysis, and comprehensive understanding [54, 55].

Naive reading involved immersing oneself in the entire study with particular emphasis on the verbatim quotes to grasp their meaning [55]. To do this, the authors read and re-read each included study and noting their own experiences as migrant African nurses in a reflective diary. Following several rounds of reading each study, the authors highlighted all quotes reflecting the phenomenon been explored. Subsequently, all verbatim quotes reflecting on transitioning or career progression in the included studies and their interpretations thereof by the original authors were transferred to a separate word file (see Table 2). The authors reflected on these quotes and the original authors’ interpretations and documented their understanding of the text. Following this, the authors undertook the thematic structural analysis which entailed explaining the text [55]. With this, the authors had in mind the question “what is the transition experience and career progression like for migrant African nurses” which enabled them to navigate through the quotes to explain the phenomenon. Codes were formulated across the quotes as meaning units [55]. These authors read, re-read, and reflected on the codes to condense similar codes. Codes with similar meanings were grouped further to generate subthemes. These were reviewed again, and similar meanings were noted by the authors. Following another round of reading, re-reading, and reviewing to attain a comprehensive understanding, similar subthemes were aggregated to generate higher-order themes which formed the basis of undertaking a narrative synthesis (see Table 2). At the end of the synthesis, the authors discussed the emerging themes. Verbatim quotes from the primary studies are provided to support the interpretation attained from the synthesis.

**Table 2:** Data extraction and synthesis

Author/ year/ setting	Study aim	Study design/ participants	Verbatim quotes	Subthemes	Themes
Aboderin, 2007 [47] United Kingdom	To presents evidence on the contexts, circumstances and perspectives of Nigerian trained nurses working in the UK and examine their relationships to globalization by building on prior analyses that use Bauman's concepts of ‘global’ and ‘local’ perspectives	Exploratory qualitative study Nigerian trained registered nurses working in the independent nursing home sector in England (*n* = 25) and registered nurses, nursing tutors and returnee migrants in Nigeria (*n* = 7)	If the economy were improved in Nigeria, we wouldn't leave our country, you understand me? That is why we came here…to adapt ourselves to the situation and then to endure. After endurance we go back home In a nursing home, you do…maybe 30% of what a nurse actually does. And it is the same every day…but…at the hospital, you meet challenges…and you do what you have been trained to do Those white…carers. They tell me ‘I have changed this man, can you change the pad’. Do you get it? I tell them ‘you as the carer are not supposed to tell me that, I am supposed to tell you that I have changed [X], can *you* change the pad Well, at the end of the day, they are white and they will side with the carers I am here for a purpose I…will go when I am ready to go I know but if that is the sacrifice I have to make I am ready to do it	Survival strategy Navigating a new culture Standing up for oneself Prejudices Survival strategy Survival strategy	Navigating reality shock Navigating reality shock Finding one’s feet Discrimination and limited opportunities for promotion
Alexis et al., 2007 [48] United Kingdom	To explore, describe and develop a greater understanding of the experiences of overseas black and minority ethnic nurses in the National Health Service (NHS) in the south of England	Phenomenological study Non-white participants originated from Asia, Africa and the Caribbean	Some relatives would by-pass me and look for a white nurse to enquire about their family member although I am the one caring for that patient We are just careful of what we have to say you know what I mean. They are permanent here and no matter what happens they can stay here but we are temporary nurses here and if we say something that they don't like we could be thrown out of the country	Preference for White over Black Prejudice/ navigating a new culture	Discrimination and limited opportunities for promotion Discrimination and limited opportunities for promotion
Henry, 2007 [49] United Kingdom	To explore the perceptions of career progression in the NHS of a group of midwives and nurses trained in Ghana and working in the UK	Qualitative case study Older Ghanaian nurses	she got it (promotion) because the questions they asked and everything, it's her natural language, that's her natural expression … for the African you have to prod … because that is the way we do things, you know. So the panel who are not used to the Africans, their way of life, or mannerisms, they say that we are thick, we cannot perform. But we can perform, we've got it all here, but sometimes it's the expression to make the panel understand us or to impress them … (she) looked at the job specification and then she started telling me ‘you know this, this, this’ … but the way she put it was not how I would have put it … she knew the buzz words she knew the words the people, the panel want they tell us to sell ourselves, but then what are we saying to sell ourselves? What should we say to sell ourselves? (It) is our downfall because we don't know what to say and this is a time that we need help. How do you sell yourself … We need somebody to tell us that this is how the panel want it and then we will say it because we've got it there. But it's not forthcoming It's not information I do not have, it's how to structure the information … how to make it sensible to me and to understand it and to be able to deliver it in the acceptable way. That is all we need … But that is what we don't get, but that is why, especially the African group of midwives and nurses, we don't move forward you hear the manager or some senior member of staff call a colleague who is also going for the interview, they go to office for a long time and they come back they are doing this (and) that … (but) nobody even asks me …’'have you prepared or is somebody helping you prepare for the interview?’’ No-one, no senior member of staff ever called me or asked me ‘they would call people to their offices and show them, tell them everything’ what was happening at the time was that they would help other people because I witnessed so many other things in that they would call some people Just before the interview I asked (the manager) if I could come for coaching, she said ‘‘no, no I may be on the panel so I won't be able to help you’’ so the day before the interview all she could do was just help me with how to use the projector … but the manager called my colleague to her office and rang personnel, got her an application, helped her to apply for a G grade I didn't get that kind of help. I would go to them and then … present myself and ask them what they think about the issues that were going to be interviewed on and then I tell them how I think it is. I tell them my version and they tell me it is alright. So I go away thinking that what I know is alright and is enough to go through the interview. So I go to the interview I get asked the questions and I tell them what I know and then it's like I make a fool of myself I've seen it and I've heard about it. I've seen it and I've sensed it. That this is what they do, they make arrangements with them, they go, they show them structure, ‘‘this is what you say, this is what you say, this is what you say’’. Now we don't get that sort of help. That's why we go for interviews and we don't pass We are afraid to make noise. That is what a lot of the black and ethnic minority staff are going through because they will be victimised … you will be victimised in such a way that nothing will move on for you. You will be noted as a trouble-maker and because of that people would rather suffer in silence (and) get their daily bread … we are not only looking after ourselves and our immediate dependents, we also have an extended family and we need to view that, who needs an enemy at your job place	Lack of recognition/ prejudice Prejudice Limited opportunities for workplace promotion Prejudice/ limited opportunities for workplace promotion Discrimination Discrimination Discrimination/ limited opportunities for workplace promotion Navigating a new culture	Discrimination and limited opportunities for promotion Discrimination and limited opportunities for promotion Discrimination and limited opportunities for promotion Discrimination and limited opportunities for promotion Discrimination and limited opportunities for promotion Discrimination and limited opportunities for promotion Discrimination and limited opportunities for promotion Discrimination and limited opportunities for promotion Discrimination and limited opportunities for promotion Navigating reality shock
Iheduru-Anderson & Wahi, 2018 [50] United States of America	To characterize the facilitators and barriers to transition of Nigerian IENs (NIENs) to the United States health care setting	Descriptive phenomenology Six Nigerian nurses (all females) with an average duration of stay in the United States at the time of the study of 14.5 years, and an average age of 39 years The average number of years they spent as RNs was 18, and average years of experience as nurses in the United States was 11	I was afraid to ask questions... I was kind of scared, I didn’t know the right thing to do. I was just dying inside and getting frustrated They did not even give me a chance, they judged my competency and knowledge based on my accent and skin color The nurses I worked with there were horrible. They were insensitive, they acted like they did not want me there.... They gang up on me and they were always reporting me to the manager, for no apparent reason.... Overall, it was a hostile environment They did not accept me in that place. You can’t just exclude the foreign trained nurses and judge them by the language, that they had an accent... [The patient] said, “Could you please get someone who speaks English because I am not even sure where you got your nursing license [In my previous job] they offer no support, even the preceptors. But in my second job, they gave me a good orientation, and I had support from another staff member who went out of her way to really give me more, and I was comfortable asking questions and she really helped me out and that made it more comfortable for me to work in that place Hard work, determination. I am not a quitter, I still have an accent but I have learned to speak so they can understand me better My nursing in Nigeria really prepared me well for my nursing practice in the US. They really did prepare me well to function as a nurse anywhere. I passed my board exams with no problems I had people I already knew that were working there. You know, other foreign educated nurses from Nigeria. I took the second job based on their conviction and assurance. They supported me all the way. I think because of them I am practicing as a nurse today. Having the other nurses from West Africa made a huge difference. I felt like I belong You just have to work hard in life and you can make it. Don’t give up... Don’t let other people issues get in your way. When all is said and done, we are better off here than at home [in Nigeria]. There is so much you can do here [in the US] as a nurse. So many places you can work. There are lots of options and opportunities for nurses here than back home. So it is better here I like the specialties here because, you specialize in one thing, this is your area and you know it well. That’s better than being everywhere. I prefer the specialties I will tell other Nigerian nurses the same thing, you will get used to it, just don’t let it bother you, don’t let them break you. It is just a phase, we all go through Because of all the bad experiences I had when I first started nursing in the country, what I can say is that I have grown professionally and otherwise... So to think of the impact it has made on me as a person, it has allowed me to understand what their perception of me is, and their perception of me as a professional nurse I believe a transitional program [would help], where foreign trained nurses can do like a three to six months training in a hospital	Navigating a new culture Navigating a new culture Navigating a new culture Navigating a new culture/ prejudice Survival strategy and support amidst shock Experiencing growth Experiencing growth Support amidst shock Experiencing growth Experiencing growth Experiencing growth Experiencing growth Support amidst the shock	Navigating reality shock Navigating reality shock Navigating reality shock Discrimination and limited opportunities for promotion Navigating reality shock Finding one’s feet Navigating reality shock Finding one’s feet Navigating reality shock
Jose, 2011 [51] United States of America	To elicit and describe the lived experiences of internationally educated nurses (IENs) who work in a multi-hospital medical centre in the urban USA	Phenomenology IENs who had migrated from the countries of the Philippines, Nigeria and India to the USA	‘You know. The idea was once you get to America, you can make it. [without] any hindrances or difficulties’ ‘I feel if the families are there, they show love and concern by doing things for the patient and making him ready to go home’ ‘I had to learn to say things in a way they will understand’ I love the profession. I think America is a good place for me to do a better job and to expand in my education and practice’ ‘It's just that it's hard to come, pick up the language, the culture, in a short time. You've got to go slow. We are good nurses with lots of potential, but it's just that everything's overwhelming [in the beginning]’	Navigating a new culture Support Experiencing growth Navigating a new culture	Navigating reality shock Navigating reality shock Finding one’s feet Navigating reality shock
Larsen, 2007 [52] United Kingdom	To examine empirically and in-depth how discriminatory attitudes and practices are experienced by overseas nurses and how the discrimination may affect their well-being and career progression	Phenomenology Two migrant nurses	We felt what we were being trained for was really not nursing, not the kind of thing we were going to go into after[wards]. It was more carers’ work, like an extra hand. We're actually paid as well and rota'd with the carers [W]e were working as carers with them [the carers]. The next thing people expected a lot more from you. You get attitudes which isn't very nice towards your work and everything. … you know that negative attitude, like ‘who's done this?’ and ‘who's whatever’ and ‘these are the nurses’ and that kind of thing [N]ot accepting you as you are, that's how I felt about it. It was like, ‘these Black nurses, what do they know? Are they really nurses professionally or what?’ NHS now, things are different, they are not so bad as they were in the nursing home. But, you see, some of the things, you say to yourself: ‘what's happening here, what's this all about?’… A visitor comes, someone who's not knowledgeable, instead of talking to the staff nurse, who's there, about the patient, they'd rather talk to the student nurse, because [she is] White and you are Black. …You get that sort of thing on and off from different groups of people. It could be the patient or the visitor. It could be colleagues He was telling me that ‘if you don't finish your two years, you have to stay here for two years or you go back to your country.’… And I was the only coloured person where I was working. My first problem I had with the Matron was a clinical issue, was a nursing issue. The way we were trained [in Nigeria], the medical language, we knew everything. But [in Britain] they don't seem to understand anything. So when you say something they say ‘you know too much’ or ‘you are arrogant’. … She was sending this carer and trained nurses after me. When I'm on nights, I'm meant to start the medication at 10 o'clock. By 9 o'clock someone's coming in and telling me to give the medication. I'm telling ‘it is not yet time for me to start’. Even the people working there said ‘how did you get yourself registered?’. When they asked me several times I said ‘I stole it, can you please call the police’ Of course! The discrimination started from that salary. A carer who cannot be earning more than that, then it's discrimination. Apart from that, they were looking for every fault. They would phone the matron by 9·30 to say I hadn't started the medication. The matron would drive eight miles to come and spy. At night, in narrow roads! Eight miles! Before she got there I must have started something. She'd walk in and go round. I got to know that they were actually setting traps Yes, they didn't want me around. There was a time they sent a letter to the health authority, unfounded allegations. The reply came and they said ‘these are internal things, not professional’ From being very, a very confident person and like somebody who's outspoken and going to say what's going to be said, I sort of drew in, into a shell, just to protect myself. You know what I mean? You really don't know where you stand, you've made your choice, you want to go on with what you've done, you weigh the pros and cons. If I really do start questioning and all that, ‘oh this nurse, she's like that’ but you want things to be done in the right way. You are not really sure who to go to. And who to trust… Actually, it's knocked my confidence outright. I don't think I've gained my confidence at all up to now. I don't think so I've struggled since I came up to now. I made some personality changes and have adapted. I will leave it at that. I'm comfortable, I don't want to create any problems. I think for the time being I'll leave it as it is. That's how I feel about it I'll leave it like that for the time being. I'm contented with what I have. If I become an E grade nurse I'll have extra responsibilities. I'm not ready for that at all. The experience I've had I do want my career to develop, but at the moment in time I still don't know what I shall do next. I don't know. I want to move out of the [specialist] ward and get another experience in another hospital. I would like [to]. [But] when you think of all the experience, getting to know new people, different attitudes again and all that. I don't think I can stand it again at the moment [I]f you talk about discrimination, you'll never move. If you come into [a] room yourself, nobody can discriminate against you. That is one thing I realised. So I don't count on it Because you'd be depressed. Because whatever they do, even if it is not discrimination, you would think ‘oh God, that again!’. You understand? Kind of misinterpretation of peoples’ actions and you would just be rejected every time Yes. It stops you, believe it or not. If I was thinking of discrimination before interviews then I will have decided within me that I will never apply there again. Then I will have been stopped. I did not think about that and I continued. And that's how I got this job \\Yes. If you know your stuff, there are people that will see that ‘this guy knows what he's talking about, his knowledge, experience’. … They are distractions. Even if you live in the midst of discrimination, if you want to move you [inaudible], but if you live and you focus on discrimination… because you always get it every day. Or you misinterpret everything to be it every day. Then you'll always be rejected and feeling sad. But if you look at ‘where do you go, this is where I'm headed’, get yourself focused	Navigating a new culture Prejudice/ discrimination Prejudice/ discrimination Prejudice/ discrimination Prejudice Discrimination Prejudice/ discrimination Navigating a new culture Navigating a new culture Navigating a new culture Navigating a new culture Limited opportunities for workplace promotion Experiencing growth Experiencing growth Experiencing growth Experiencing growth	Navigating reality shock Discrimination and limited opportunities for promotion Discrimination and limited opportunities for promotion Discrimination and limited opportunities for promotion Discrimination and limited opportunities for promotion Discrimination and limited opportunities for promotion Discrimination and limited opportunities for promotion Navigating reality shock Navigating reality shock Discrimination and limited opportunities for promotion Finding one’s feet Finding one’s feet Finding one’s feet Finding one’s feet
Likupe, 2015 [26] United Kingdom	To explore experiences of racism, discrimination and equality of opportunity among black African nurses and their managers' perspective on these issues	Qualitative study Thirty nurses from Malawi, Kenya, Ghana, Nigeria, South Africa, Zambia, Zimbabwe and Cameroon, had been in the UK between two and five years and were all at staff nurse grade Nurses had experience ranging from five to 20 years in their countries of origin. Two nurses had been lecturers in their country of origin while the rest had been sisters and staff nurses	‘Nobody recognises any black (nurse) no matter how intelligent you are. If you are intelligent they would rather prove you to be too confrontational. So I tell you I cannot hide, I told my manager last week I said I'm not happy’ ‘When the Indian nurses were recruited there was this thing (policy) about recruitment and retention so you find that the kind of treatment that they were given is that kind of treatment that would want to keep people where they are and then after a while it (the feeling that Indian nurses were recruited to be retained) all worn off and that's why they came up with this attitude that they are better than you because they were given the impression that they were needed more than you are’ ‘We had one Hungarian lady who was working with us but you won't believe this lady was treated differently. We were saying the work she was doing wasn't the right thing but she was treated like… you know because of the colour as if she knows what she is doing’ ‘With my senior colleagues there was of course that feeling that I was under scrutiny all the time and it took time for them to understand that I can do the same things they do just as well as they do’ ‘Some of the residents accepted me but some were not happy to be looked after by a black person and I was told by the manager that room 40,room 43 and room 18 you should not go there because they don't like to be looked after by a black person’ ‘Even relatives, they see that you are the nurse; they have seen your badge, because we have similar uniform with the health carers but you can still see the difference. If they are enquiring something about their relative, they will bypass you, even if you are the first person they see, they will go to the health carer and then the carer will say no you go to that one, that's when they will come’ ‘…someone had chest pain and we had to do an ECG and I did it because I know how to do it although there is a pack that you should have which I don't have. She (the ward manager) said what you did was wrong and you don't have the pack so don't do it again. I said okay. She called an Indian lady to do it and she said sister but I don't have a pack as well and the sister said go and do it. I said what you are doing is discrimination’ ‘The best word I can use is racism or discrimination, as long as you come from Africa you are not one of them, you are not a white person, you are looked down upon in every way, there is racism, even when you are in a meeting, like making a suggestion, they won't take it into account because to them you are black and you don't know anything, you know, that's the thing’ ‘I have been there more than a year now, but there was a white nurse who came to work there after finishing her training, she just worked for six months and now she has been promoted to E grade. And you can imagine what impact it has on us’ ‘There are times when I have asked to go for a course, like me and another person want to go for the same course, they will choose their own people. We should feel that we've got the same qualifications and we contribute the same skills like white nurses and I think they should put equal opportunities in practice otherwise its of no use’	Lack of recognition Prejudice/ lack of recognition Prejudices Navigating a new culture Prejudices Preference for White over Black Discrimination Discrimination/ prejudices Discrimination/ prejudices/ limited workplace promotion Limited workplace promotion	Discrimination and limited opportunities for promotion Discrimination and limited opportunities for promotion Discrimination and limited opportunities for promotion Navigating reality shock Discrimination and limited opportunities for promotion Discrimination and limited opportunities for promotion Discrimination and limited opportunities for promotion Discrimination and limited opportunities for promotion Discrimination and limited opportunities for promotion Discrimination and limited opportunities for promotion
Likupe & Archibong, 2013 [27] United Kingdom	To explore Black African nurses’ experiences of equal opportunities, racism, and discrimination in four NHS trusts in northeastern England	Qualitative study Thirty nurses from sub-Saharan countries working in four NHS trusts; Thirty nurses from Malawi, Kenya, Ghana, Nigeria, South Africa, Zambia, Zimbabwe and Cameroon, had been in the UK between two and five years and were all at staff nurse grade Nurses had experience ranging from five to 20 years in their countries of origin. Two nurses had been lecturers in their country of origin while the rest had been sisters and staff nurses	It could be anybody from Hungary, the Philippines it could be someone, but because you are coming from Africa there’s lack of respect. We are all professionals trained and if someone comes to a ward and doesn’t know the ward, the way it works, obviously the person will ask some questions. Africans are treated not nicely at all We had one Hungarian lady who was working with us, but you won’t believe this lady was treated differently. At the end we say the work she was doing wasn’t the right thing. but she was treated like... you know, because of the color, as if she knows what she is doing It’s really bad. You feel pain that a colleague of yours from home is being told that they need to look for a job elsewhere because they are not catching up! When you know back home, you and me, what do we do? You have no doctor around, but you have to go through a hundred patients, IVs and everything, then how come the same person cannot function? Th ere has to be something wrong, and then it will affect the quality of work that they give out. Somewhere so I think they need to know that and they need to know that we are smart like anybody else I worked on this ward, the sister sat down with me at my appraisal and I said this is what I can do, I know how to do it, if you need documents, I can try to get them for you. She never believed [that I had those skills] It’s like that because we as Africans, we are not like Filipinos and Indians who are very gullible because we fi ght back, they don’t like that. And they term that as arrogant Here it’s the auxiliaries who run the wards, not managers, I tell you. If the auxiliaries don’t like you, you are fi nished. ‘Cos they say things to your manager and the manag-ers take them seriously. If I’m on night duty and my colleague goes on break, I should not get instructions from an auxiliary You could read about carers back home, you cannot really explain what carers are; you may not be using the word carer back home. We use the word ward assistant. And then in shift planning you have to come across that carer. But how do you explain who a carer is, but if you are here, where the textbook is written, you know who a carer is and you can really apply what you are reading to your environment I once gave an example to one of the nurses, I said you have been a nurse here for some time, if I take you home and just dump you in my ward, would you be able to perform the way you have been performing? She said no, you must be very courageous to come here. But, you see, instead of giving us support even to show us, but people look at us if you fail to operate a hoist [a device for lifting patients] I had some problems with them [auxilia-ries] but I just tell them I am the registered nurse, you are the auxiliary therefore you are under my instruction. I don’t care if I am Black or what, but I am the registered nurse Again, most of them don’t know where we are coming from and what we have learned. For those who have worked outside they have a lot of respect. When you work with them you can tell the diff erence from those who have always worked here. I think it depends on their exposure and their culture. Th ose who are exposed to other cultures are diff erent. Th ose who are not exposed, I guess, you can’t blame them Th e most diffi cult age group I would say are the elderly because those are the people who have never even seen the Blacks. Because in their time there were only Whites, it’s only now that there are a lot of Blacks around It was very strange because I didn’t know what to do. One of the residents became poorly and the care assistant told me so-and-so is poorly in room 18. When I went, she refused for me to get into the room, so I had to call the ward manager to come and sort her out and take her to hospital, so it was very difficult Someone had chest pain and we had to do an ECG [electrocardiogram] and I did it, because I know how to do it, although there is a pack that you should have which I don’t have. She said what you did was wrong and you don’t have the pack, so don’t do it again. She called an Indian lady to do it and she said, “Sister, but I don’t have a pack as well, and she said go and do it.” I said, “What you are doing is discrimination.” As long as you come from Africa, you are not one of them, you are not a White person, you are looked down upon in every way. Th ere is racism, even when you are in a meeting, like a suggestion, they won’t take it into account because to them you are Black and you don’t know anything I don’t bother telling anyone because I do not think that people can listen to me. You don’t have a sense of belonging	Prejudices Prejudices Prejudices Prejudices Navigating a new culture Standing up for oneself Prejudices Prejudices Prejudices/ preference for White over Black Discrimination Prejudices/ discrimination Navigating a new culture	Discrimination and limited opportunities for promotion Discrimination and limited opportunities for promotion Discrimination and limited opportunities for promotion Discrimination and limited opportunities for promotion Discrimination and limited opportunities for promotion Navigating reality shock Finding one’s feet Discrimination and limited opportunities for promotion Discrimination and limited opportunities for promotion Discrimination and limited opportunities for promotion Discrimination and limited opportunities for promotion Discrimination and limited opportunities for promotion Navigating reality shock
Okougha & Tilki, 2010 [53] United Kingdom	To explore the experiences of nurses recruited from Ghana and Philippines by a London NHS Trust	Qualitative study Nurses recruited from Ghana and Philippines	‘When I first came to England, I did not understand when a patient asked me where the “loo” was. The word “loo” is not used in my country, we use toilet.’ ‘But I fi nd out the hard way that speaking eye to eye with someone without even a blink is the British way of communication. This is usually taken that someone is a good listener or actually cares.’ ‘“Please” and “thank you” is a way of showing politeness in British culture though it is not something we do in our culture.’ ‘I can say something to my colleague and, if said in a polite way, there is no need to say “please”. Being polite does not rest in the word “please”. Some people even say “please” and it is an insult.’ ‘You listen to the intonation … In my dialect, please is built into the statement. I was brought up to ask for something in a certain way that demonstrates politeness.’ ‘You can sit [as opposed to stand] even if the director of nursing is talking to you.’ ‘When I started, my mentor was an older person professionally and in age. I called her sister. Her response was “don’t sister me”! I was shocked and said “Oh sister, it is diffi cult for me to call your name because where I come from it is an insult to just call someone older than you by fi rst name.’ ‘It is easier with colleagues but with patients I still call Mr or Mrs. In Ghana, fi rst names are not used to address or refer to older family members.’ ‘Back home, when you are addressing somebody older than you, you add a title to the name like mama, auntie, sister etc.’ ‘Coming from cultural backgrounds where your physical presence is a way of demonstrating care and concern, it is diffi cult to understand why a nurse should be chasing up relatives.’ ‘People who have parents and grandparents take care of them. But this is not the same here. We do not send our aged people to nursing homes.’ ‘Some will even go away on holiday and send a postcard! I can never do this to my mother. If my mother is sick, I cannot travel. You have to be there irrespective of how important your holiday may be.’ ‘When your relative is dying back home, you don’t go to the hospital. The staff deal with it. But here, immediately the patient’s condition changes, you call the family, the next of kin.’ ‘We [Ghanaians] are very close and, when somebody is dying, the grief that we have, you don’t want to go near. But here, they even want to spend time with the dead body before it goes to the mortuary.’ ‘Offering a cup of tea to someone who has just lost a loved one was a challenge initially.’ ‘We believe in the existence of God, so we approach things differently. Here, when you admit a patient, you ask is if they need a priest. Some want the priest to come, but the majority don’t believe in God. Back home in the hospital, we do a little prayer before we start our job for the day, but here it is not like that.’	Navigating a new culture Navigating a new culture Navigating a new culture Navigating a new culture Navigating a new culture Navigating a new culture Navigating a new culture Navigating a new culture Navigating a new culture Navigating a new culture Navigating a new culture Navigating a new culture Navigating a new culture Navigating a new culture Navigating a new culture Navigating a new culture	Navigating reality shock

### Methodological rigor

This study focused on interpreting the verbatim quotes presented in each primary study. Although the authors are all migrant nurses from Africa, we documented our experiences in a reflexive journal and through member checking. This action ensured that our experiences helped us to understand those reported in the primary studies, instead of our experiences overshadowing those reported. Additionally, each member of the research team has graduate-level training in qualitative methods which helped to undertake the study in a transparent manner while adhering to methodological principles. Following the completion of the synthesis, the findings were discussed in a webinar involving migrant African nurses. The participants affirmed that the findings reflected on their experiences as migrant nurses.

## Results

### Study characteristics

The extensive search yielded 139 studies of which nine (9) studies met the inclusion criteria for retention in this study [24, 25, 47–51, 53, 56]. All the studies received an overall appraisal “include”. However, the included studies lacked information regarding researcher positionality and reflexivity, that is, it remains unclear the assumptions that the researchers held regarding the phenomenon and how these may have impacted how they navigated the research. The studies were published from 2005 to 2018 with three of the included studies focusing solely on Nigerian migrant nurses [47, 53, 56] and one focusing solely on older Ghanaian migrant nurses [48].

Although the clinical background of the participants was not highlighted across all the included studies, participants in three of the studies comprised of both clinical nurses and nurse educators who were taking on clinical roles in their new settings (Aboderin, 2017; Likupe & Archibong, 2013; Likupe, 2015). All participants in the included studies were experienced nurses with more than 5 years of working experience in their countries of origin. More females than males participated in the primary studies.

### Themes and subthemes

Three themes and six subthemes emerged from the included studies (Table 3). The transitioning experiences of migrant African nurses were captured as involving navigating a reality shock at the initial phase albeit with some form of support for some migrant nurses. Although the migrant African nurses also experienced prejudices and discrimination as they transitioned, overtime, they experienced growth and looked beyond these setbacks. In terms of career progression, the migrant African nurses experienced limited opportunities for workplace promotion and a general lack of recognition.

**Table 3:** Themes and subthemes

Themes	Subthemes
Navigating reality shock	1. Navigating a new culture 2. Survival strategies and support amidst the shock
Discrimination and limited opportunities for promotion	1. Prejudices and preference for White over Black 2. Lack of recognition and limited opportunities for a workplace promotion
Finding one’s feet	1. Standing up for oneself and looking beyond discrimination 2. Experiencing growth

#### Theme 1: Navigating a reality shock

The theme describes the discrepancy between what the migrant nurses expected before joining the new setting and what they experienced as well as the support they received. This theme reflects the initial phase of transitioning for the migrant African nurses. The subthemes are 1) navigating a new culture, and 2) survival strategies and support amidst the reality shock.

##### Navigating a new culture

This subtheme describes the initial reality of experiencing a new culture as against what the migrant nurses were familiar with. Entry and transitioning into a new setting or workplace brought the migrant African nurses face to face with a new culture [47]. Seemingly, their notion prior to arriving at the new setting was one of a smooth process without challenges or hindrances [50]. The new workplace culture, however, differed from what they were used to back in their home countries, and as such required them to adapt which was challenging within a short timeline [47, 50]. Subtle unwritten professional codes such as demonstrating respect to senior colleagues by standing when talking to them or addressing them by their professional titles were reportedly insignificant in their new workplace but were extremely significant in their previous settings [56]. Switching suddenly to address people by their first names evoked an unpleasant feeling for the migrant African nurses as they felt it was a sign of disrespect or an insult [47, 56]. This was particularly more challenging when dealing with older colleagues at the workplace who preferred to be addressed by their first names rather than professional titles [47, 56]. In fact, the initial phase of transitioning was overwhelming and chaotic for the migrant African nurses as they navigated a new culture and engaged with a new reality [47, 50]. Below are some quotes:

“You know. The idea was once you get to America, you can make it [without] any hindrances or difficulties.” [50]

“You can sit [as opposed to stand] even if the director of nursing is talking to you. When I started, my mentor was an older person professionally and in age. I called her sister. Her response was “don’t sister me”! I was shocked and said “Oh sister, it is difficult for me to call your name because where I come from it is an insult to just call someone older than you by the first name….Back home, when you are addressing somebody older than you, you add a title to the name like mama, auntie, sister etc.” [56]

“We are good nurses with lots of potentials, but it's just that everything's overwhelming [in the beginning].” [50]

For some migrant African nurses, the new work environment was experienced as hostile due to the negative attitudes of the staff [24, 25, 47, 50]. These migrant nurses did not feel welcome and felt as though they were threats and targets for no reason which contrasted with the supposedly friendly nature of the workplaces in their home countries [24, 25, 47, 50, 51, 53]. This made them focus on their only purpose for migrating which is to work and improve their financial situation [53]:

“I am here for a purpose I…will go when I am ready to go. I know but if that is the sacrifice I have to make I am ready to do it” [53]

“The nurses I worked with there were horrible. They were insensitive, they acted like they did not want me there. They gang up on me and they were always reporting me to the manager, for no apparent reason. Overall, it was a hostile environment.” [47]

The variations in the workplace culture, patient care processes, family dynamics, and family engagement were also sources of reality shock for the migrant African nurses [24, 25, 53, 56]. Additionally, the nurses were shocked to identify patients who did not belong to any religion or who did not believe in the existence of God which contrasts with the religious climate in their home countries [56]:

“Offering a cup of tea to someone who has just lost a loved one was a challenge initially. We believe in the existence of God, so we approach things differently. Here, when you admit a patient, you ask if they need a priest. Some want the priest to come, but the majority don’t believe in God. Back home in the hospital, we do a little prayer before we start our job for the day, but here it is not like that.” [56]

Language also emerged as another shock for migrant nurses [51, 56]. Although the migrant African nurses were proficient in the use of written and oral English, they still found it challenging to understand the slangs and non-verbal cues used in their new settings [51, 53, 56]. This made it rather difficult to occasionally communicate and understand the needs of their patients, particularly at the initial phase of their transitioning to the new setting [47, 56]:

“When I first came to England, I did not understand when a patient asked me where the “loo” was. The word “loo” is not used in my country, we use toilet” [56]

##### Survival strategies and support amidst the shock

The subtheme describes how the migrant nurses survived through the initial shock as they transitioned. To survive, some migrant African nurses maintained a culture of silence so as not to appear as troublemakers which they felt can adversely impact their work, and affect their financial situation [47, 48, 53]:

“We are afraid to make noise. That is what a lot of the black and ethnic minority staff are going through because they will be victimized … you will be victimized in such a way that nothing will move on for you. You will be noted as a trouble-maker and because of that people would rather suffer in silence (and) get their daily bread … we are not only looking after ourselves and our immediate dependents, we also have an extended family and we need to view that, who needs an enemy at your job place.” [48]

Although support structures were generally limited, some migrant African nurses highlighted the support they received as they transitioned to their new workplace. Peer support from some colleagues at the workplace and other persons from their home countries residing in their new setting were particularly helpful as they transitioned to the new place [47, 48, 53, 56]. Such sources of support made the migrant nurses feel a sense of belongingness [47]. Facility-based transitioning programme was highlighted in one study which the participants described as helpful as they transitioned to their new setting and roles [47]:

“[In my previous job] they offer no support, even the preceptors. But in my second job, they gave me a good orientation, and I had support from another staff member who went out of her way to really give me more, and I was comfortable asking questions and she really helped me out and that made it more comfortable for me to work in that place…I had people I already knew that were working there. You know, other foreign educated nurses from Nigeria. I took the second job based on their conviction and assurance. They supported me all the way. I think because of them I am practicing as a nurse today. Having the other nurses from West Africa made a huge difference. I felt like I belong.” [47]

#### Theme 2: Discrimination and limited opportunities for promotion

The theme describes the prejudicial treatment experienced by the migrant African nurses as they transitioned to their new settings and the limited opportunities for career progression they faced. The subthemes are 1) Prejudices and preference for White over Black, and 2) Lack of recognition and limited opportunities for workplace promotion.

##### Prejudices and preference for White over Black

Migrant African nurses felt they were racially discriminated against when compared to domestic nurses or foreign-trained nurses from other countries across all included studies [24, 25, 47–51, 53, 56]. The migrant nurses highlighted how patients preferred to have White nurses take care of them instead of the African nurses with the notion that the latter category of nurses was not up to the task or could not communicate well [24, 25, 47–51, 53, 56]:

“Some of the residents accepted me but some were not happy to be looked after by a black person and I was told by the manager that room 40, room 43 and room 18 you should not go there because they don't like to be looked after by a black person.” [24]

Additionally, other healthcare practitioners and family members often chose to interact more with the White nurses (irrespective of their professional status) rather than the African nurses [24, 25, 47, 56]. At the initial phase of their transitioning experience, the migrant African nurses felt they were constantly under surveillance as some colleagues doubted their skills and professional capacity albeit overtime, the scrutiny diminished [24, 25, 47]. These experiences made the migrant nurses feel as though they did not belong with a sense of exclusion [24, 25, 47, 49, 51]:

“Some relatives would by-pass me and look for a white nurse to enquire about their family member although I am the one caring for that patient.” [51]

“They did not accept me in that place. You can’t just exclude the foreign-trained nurses and judge them by the language, that they had an accent . . . [The patient] said, “Could you please get someone who speaks English because I am not even sure where you got your nursing license” [47]

“With my senior colleagues, there was of course that feeling that I was under scrutiny all the time and it took time for them to understand that I can do the same things they do just as well as they do.” [24]

Migrant African nurses who worked in care homes felt under-utilized and could not employ their broad range of skills as Registered Nurses [53]. This was observed to be related to their sole involvement in basic, routine care on daily basis. Besides, these routine tasks were not considered to be challenging [51, 53]:

“In a nursing home, you do…maybe 30% of what a nurse actually does. And it is the same every day…but…at the hospital, you meet challenges…and you do what you have been trained to do.” [53]

Migrant African nurses were looked down upon irrespective of their efforts and not respected [24, 25, 47]. In fact, the colour of the migrant nurses formed the basis of how they were treated [24, 25]:

“The best word I can use is racism or discrimination, as long as you come from Africa you are not one of them, you are not a white person, you are looked down upon in every way, there is racism, even when you are in a meeting, like making a suggestion, they won't take it into account because to them you are black and you don't know anything, you know, that's the thing.” [24]

“It could be anybody from Hungary, the Philippines it could be someone, but because you are coming from Africa there’s a lack of respect. We are all professionals trained and if someone comes to a ward, and doesn’t know the ward, the way it works, obviously the person will ask some questions. Africans are treated not nicely at all”. [25]

Even when it came to clinical duties, migrant African nurses felt they were discriminated against and in most instances, the White nurses may be asked to attempt a task before it was assigned to them [24, 25, 47, 56]:

“…someone had chest pain and we had to do an ECG and I did it because I know how to do it although there is a pack that you should have which I don't have. She (the ward manager) said what you did was wrong, and you don't have the pack so don't do it again. I said okay. She called an Indian lady to do it and she said sister but I don't have a pack as well and the sister said go and do it. I said what you are doing is discrimination.” [24]

##### Lack of recognition and limited opportunities for a workplace promotion

The existence of racial discrimination and prejudices were observed to further permeate the opportunities available to migrant African nurses with a general lack of recognition of their efforts as professional nurses [24, 25, 48, 50, 51, 53]:

“Nobody recognizes any black (nurse) no matter how intelligent you are. If you are intelligent, they would rather prove you to be too confrontational. So I tell you I cannot hide, I told my manager last week I said I'm not happy.” [24]

“As long as you come from Africa, you are not one of them, you are not a White person, you are looked down upon in every way. There is racism, even when you are in a meeting, like a suggestion, they won’t take it into account because to them you are Black and you don’t know anything.” [25]

Migrant African nurses felt they were disadvantaged when it came to workplace promotion or career progression with the preference of White nurses over African nurses [24, 25, 48–50, 56]. There was little-to-no form of support or guidelines to help them prepare for promotion interviews and even when they had all the information, they were unable to package and utilize the information [24, 25, 47, 48]:

“…she got it (promotion) because the questions they asked and everything, it's her natural language, that's her natural expression … for the African, you must prod … because that is the way we do things, you know. So, the panel who are not used to the Africans, their way of life, or mannerisms, they say that we are thick, we cannot perform. But we can perform, we've got it all here, but sometimes it's the expression to make the panel understand us or to impress them” [48]

“It's not information I do not have, it's how to structure the information … how to make it sensible to me and to understand it …me or asked me” [48]

“Whilst I have been there more than a year now, but there was a White nurse who came to work there after finishing her training, she just worked for six months and now she has been promoted to E grade. And you can imagine what impact it has on us.” [25]

With the challenges experienced as they worked, some migrant African nurses did not feel as though they wanted to move up the career ladder anymore [47–49, 56]. They felt the career pathway was muddled [24, 25, 48, 49]. The culture of silence persisted even after transitioning to the new setting [24, 25]:

“I do want my career to develop, but at the moment in time, I still don't know what I shall do next. I don't know. I want to move out of the [specialist] ward and get another experience in another hospital. I would like [to]. [But] when you think of all the experience, getting to know new people, different attitudes again and all that. I don't think I can stand it again at the moment… I'll leave it like that for the time being. I'm content with what I have. If I become an E grade nurse, I'll have extra responsibilities. I'm not ready for that at all. The experience I've had.” [49]

“They did not even give me a chance, they judged my competency and knowledge based on my accent and skin colour.” [47]

#### Theme 3: Finding one’s feet

The theme describes the growth experienced by the migrant African nurses despite the presence of racial discrimination and prejudices. The subthemes are 1) Standing up for oneself and looking beyond discrimination, and 2) Experiencing growth.

##### Standing up for oneself and looking beyond discrimination

As the migrant nurses transitioned further and understood their new setting, they began to stand up for themselves in subtle ways [24, 25, 47, 49, 51, 53, 56]. Though the culture of silence and discrimination may persist, they occasionally stood their grounds as professional nurses [24, 25, 47, 49, 51, 53, 56]:

“I had some problems with them [auxiliaries] but I just tell them I am the registered nurse; you are the auxiliary therefore you are under my instruction. I don’t care if I am Black or what, but I am the registered nurse.” [25]

“If you come into [a] room yourself, nobody can discriminate against you. That is one thing I realized. So I don't count on it.” [49]

Gradually, the migrant African nurses began to view the experience of discrimination as a distraction [48, 49]. Instead of ‘fighting’ the system, they attempted to look beyond the racial discrimination and prejudices to the opportunities available to them [47–50]:

“Yes. It stops you, believe it or not. If I was thinking of discrimination before interviews, then I will have decided within me that I will never apply there again. Then I will have been stopped. I did not think about that, and I continued. And that's how I got this job.” [49]

##### Experiencing growth

With hard work and determination, migrant African nurses worked their way through their experiences to improve their communication with the patients and other healthcare practitioners as well as move beyond their initial negative experiences [24, 25, 47–50]. They had a better understanding of their new environment and felt it was still better compared to their home situation [24, 25, 47, 48, 51, 53, 56]. They developed their coping strategies as they transitioned. They began to appreciate the opportunities available to them including varied workplaces and varied specialties [24, 25, 47–51, 53, 56]:

“Hard work, determination. I am not a quitter; I still have an accent, but I have learned to speak so they can understand me better. My nursing in Nigeria really prepared me well for my nursing practice in the US. They really did prepare me well to function as a nurse anywhere. I passed my board exams with no problems”. [47]

## Discussion

This review synthesized existing qualitative studies to develop an understanding of the transitioning and career progression experiences of migrant African nurses. Despite the limited number of studies exploring the phenomenon, this synthesis revealed that the initial transitioning experiences of migrant African nurses involved navigating several shocks and experiencing discrimination/ prejudices. Over time, the migrant nurses began to view the negative experiences as a distraction as they explored and identified ways of surviving and thriving in their new settings. Career progression pathways for the migrant nurses seemed unclear with limited opportunities for workplace promotion. Notwithstanding these experiences, the migrant nurses felt they were better off in their new settings compared to their home countries due to the numerous opportunities for personal and professional development. This gave them the impetus to remain and continue working. Overall, the review findings affirm the need for tailor-made workplace transitioning/ orientation and support programmes to help migrant African nurses to adapt to and navigate through their new setting, particularly at the initial phase. Additionally, healthcare facilities should include career planning in transitioning and support programmes for migrant nurses. The discussion will be structured under the following sub-headings: 1) opportunities for practice, 2) opportunities for research, and 3) opportunities for policies.

### Opportunities for practice

Transitioning is an ongoing process and remains unclear when this process ends [52]. Transitioning to a new setting presents opportunities for learning and growth but may also be a breeding ground for negative experiences for migrant nurses [57]. Migration to work in a new setting requires being immersed in the culture of the workplace and the wider context which warrants further attention to be paid to how they navigate the new culture, the cultural clashes that may occur, how they resolve these clashes and internalize the new culture [58]. These experiences encapsulate the experience of reality shock [59, 60]. Interestingly, despite the success of overseas recruitment drives in attracting these nurses, only limited attention has been paid to the acculturation process [5]. Thus, it remains unclear how to support the process, particularly as negative experiences can adversely impact the perceptions of migrant nurses about their new settings. The notion of reality shock has previously been highlighted among other internationally trained healthcare professionals. These studies have reported reality shock to be associated with emotional stressors related to meeting basic life necessities such as transportation and accommodation [61, 62]. Additionally, reality shock may be associated with feelings of isolation and homesickness due to the absence of family and familiar social networks [63, 64]. Although African nurses may also experience isolation, this is related to the shock of being excluded in their new settings, discrimination, and prejudices [24, 25, 47, 48, 56]. Acculturation is therefore likely to differ across the groups of internationally trained nurses [31, 65]. This finding may have implications for practice. Firstly, the findings may suggest that tailor-made transitioning and adaptation programmes may be potentially more helpful compared to generic programmes developed for all migrant nurses. These programmes need to take into consideration the unique background of the migrant nurses to prepare them for the transitioning, adaptation, and integration process ahead. Secondly, there is a need to look beyond sole recruitment and the pressure to fill vacancies rapidly to understand and appreciate the migration trajectories of the migrant nurses, their expectations, and how well they transition to their new settings/ roles [5].

Another interesting finding worth mentioning is the language barrier experienced by the migrant African nurses. Professional registration in developed countries such as the UK, USA, New Zealand, and Australia require meeting English language standards on either Test of English as a Foreign Language (TOEFL), Occupational English Test (OET), or the International English Language Testing System (IELTS) for migrants not considered as coming from a majority English-speaking country which may include most, if not all African countries [66]. Despite meeting the required language standards, a nuanced form of language barrier still exists as the migrant nurses come face to face with the slangs or jargons used in their new settings. Of course, we cannot advocate for the abolishment of the current language requirements since it assesses several aspects of language competencies. However, transitioning programmes need to incorporate additional language support to facilitate the adaptation process.

### Opportunities for policy

Racial discrimination and prejudice emerged as significant issues experienced by migrant African nurses as they transitioned. Racial and ethnic prejudice and discrimination have been described as a major issue affecting migrant nurses as one study conducted in Britain reported that more than 66% of Black nurses and more than 50% of Asian nurses were targets of discrimination by patients and family members [67]. Another study also described the behaviour of some White nurses toward Black nurses as abusive [51]. Migrant nurses are more prone to discrimination than native nurses and the situation may be worse among migrant African nurses [32, 68]. Despite this reality, the current review noted that migrant African nurses are likely to overlook racial discrimination and prejudices with a culture of silence. This is indeed worrying as remaining silent only worsens the situation. With the pace of the ongoing migration of nurses, institutional policies must be enacted to facilitate the reporting and resolution of cases involving racial discrimination, prejudice, and abuse. Healthcare institutions need to strengthen policies to effectively address this harmful practice. Additionally, institution-wide training is needed to improve working relationships and handling of issues involving racial discrimination and prejudices [69].

Career progression is another area warranting attention for migrant African nurses considering how racial discrimination and prejudices may permeate this area. A recent study that explored the nature of career progression delays for Black, Asian and Minority Ethnic (BAME) nurses in the UK observed that BAME nurses and midwives had spent more months working at the entry-level grade than White nurses and midwives and fewer months at higher grades over the previous 10 years [70]. BAME Ethnic nurses and midwives were less likely to have received professional training in the previous year and had to apply for significantly more posts than White nurses and midwives before gaining their first post in their current band [70]. In addition to racial discrimination, it has been reported that lack of access to mentorship and support often discourages most migrant African nurses from applying for high-level positions [71]. Career progression means a lot to professionals, especially in the healthcare system where there is a constant update on medical practice and nursing care. Thus, working in a setting where the career progression pathways are unclear can push healthcare workers away to other places [72, 73]. Perhaps, it will be helpful if institutions provide clear guidelines and policies regarding career progression. Migrant nurses will also need to be proactive in seeking these opportunities and support when required.

### Opportunities for research

As noted in the results section of this study, only one study exploring the transitioning and career progression experiences of migrant African nurses have been published after 2015 and this study focused solely on migrants from Nigeria [47]. This is quite worrying considering the ongoing nurse migration from the African continent. Thus, there are opportunities for further research to understand the experiences of migrant African nurses across the globe. Considering that most studies included in this review focused on nurses working in the clinical setting suggest that significant gaps regarding the experiences of other categories of nurses such as nurse educators and nurse researchers remain minimally explored. Migrant African nurses.

### Study limitations

Notwithstanding the interesting findings, some limitations are noteworthy. Most of the studies included migrant nurses from West African countries such as Nigeria and Ghana. Thus, the findings may not reflect the entire African story. More studies are needed in this regard to update this meta-synthesis. Secondly, most of the participants had migrated to the UK and USA with limited information regarding transitioning experiences from other countries. Though some primary studies were identified from Australia and Germany, these studies did not provide verbatim quotes of the African nurse participants and as such were excluded. These limitations notwithstanding, we have provided a thick description of the phenomenon based on the available data.

## Conclusion

The migration of nurses from Africa is an ongoing reality. Transitioning to a new setting can be a challenging experience warranting the availability of a tailor-made adaptation or orientation programme. Though African nurses may experience discrimination and prejudices, they consider their situation to be better off compared to their home countries. Career pathways most often seem unclear requiring institutions to enact clear policies and migrant nurses to be proactive in taking on an active role in pushing their careers ahead using opportunities created through continuous professional development and further education to position themselves for higher roles in their area of practice.

## Data Availability

All data generated or analysed during this study are included in this published article [and its supplementary information files].
